# Mechanistic basis and therapeutic modulation of T cell fitness to enhance CAR-T cell efficacy in hematological malignancies

**DOI:** 10.3389/fimmu.2026.1762453

**Published:** 2026-02-18

**Authors:** Karama Makni-Maalej, Shaykhah Mujahhiz Alotaibi, Queenie Fernandes, Syed Osman Ahmed, Sarra Mestiri, Salim Bougarn, Waad Amir, Syed Farhatullah, Mohamed Kharfan-Dabaja, Maysaloun Merhi, Riad El Fakih, Mahmoud Aljurf, Said Dermime

**Affiliations:** 1 Translational Cancer Research Facility, National Center for Cancer Care and Research, Hamad Medical Corporation, Doha, Qatar; 2 Department of Hematology, Stem Cell Transplantation & Cellular Therapy, King Faisal Specialist Hospital and Research Centre, Riyadh, Saudi Arabia; 3 Qatar Biomedical Research Institute, Hamad Bin Khalifa University, Qatar Foundation, Doha, Qatar; 4 Mayo Clinic, Division of Hematology-Oncology and Blood and Marrow Transplantation and Cellular Therapy Program, Jacksonville, FL, United States; 5 College of Health Sciences, Qatar University, Doha, Qatar

**Keywords:** CAR-T cell therapy, hematological malignancies, metabolic reprogramming, T cell fitness, T cell subsets

## Abstract

T cell fitness has emerged as a critical determinant of the efficacy and persistence of Chimeric Antigen Receptor (CAR)-T cell therapy. Defined by the capacity of T cells to proliferate, resist exhaustion, persist *in vivo*, and exert sustained effector functions, T cell fitness reflects the integration of a dynamic network of intrinsic and extrinsic regulatory mechanisms. In this review, we present a comprehensive synthesis of the molecular and cellular foundations underlying T cell fitness, emphasizing the influence of differentiation trajectories, signaling pathways, metabolic reprogramming, and epigenetic modifications. We further discuss the impact of patient-specific conditions such as age as well as disease biology, prior therapeutic exposures, and timing and quality of T cell collection, on the phenotypic and functional efficacy of CAR-T cell products. Beyond delineating these determinants, we highlight emerging strategies aimed at enhancing T cell fitness. Importantly, we propose T cell fitness as an integrated, multi-layered systems property emerging from the interaction between differentiation state, signaling architecture, metabolic–mitochondrial competence, epigenetic stability, and host-specific inflammatory and treatment-related pressures. We introduce a mechanistic framework that links these layers across the CAR-T therapeutic timeline from leukapheresis to post-infusion tumor engagement and outline how this framework can be operationalized into measurable parameters to guide patient stratification, manufacturing decisions, and rational therapeutic interventions.

## Graphical Abstract

**Figure:**
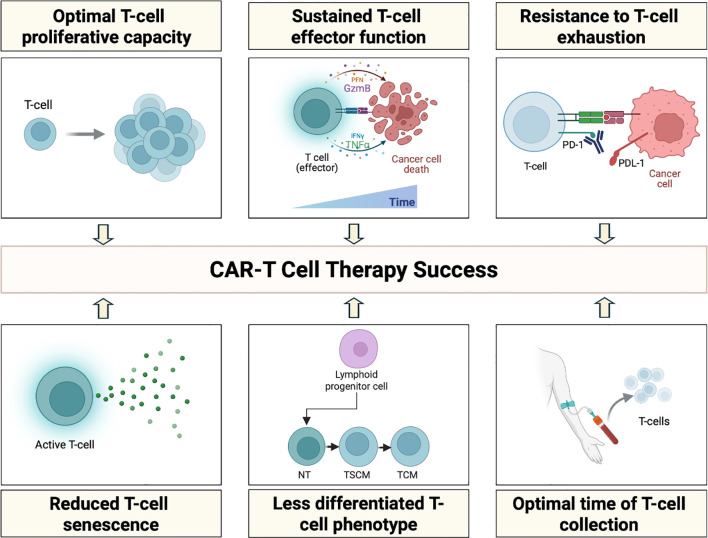

## 1 Introduction

The advent of chimeric antigen receptor (CAR)-T cell therapy has marked a significant milestone in the field of cellular immunotherapy, particularly in patients with refractory B-cell malignancies (1, 2). By genetically engineering T cells to express CARs that target specific tumor antigens, this therapy has achieved remarkable success in hematological malignancies such as acute lymphoblastic leukemia (ALL), various types of B-cell non-Hodgkin lymphoma (NHL), and multiple myeloma (MM) (3). Currently, seven CAR-T cell therapies (Abecma^®^, Aucatzyl^®^, Breyanzi^®^, Carvykti™, Kymriah^®^, Tecartus^®^, and Yescarta^®^) have been approved by the Food and Drug Administration (FDA) and are commercially available as therapies for B-cell acute lymphoblastic leukemia (ALL), chronic lymphocytic leukemia (CLL), large B-cell lymphoma (LBCL), follicular lymphoma, mantle cell lymphoma, and multiple myeloma (4, 5). Indeed, five of these therapies target CD19, which is found on the surface of healthy and cancerous B cells, and two of them target B-cell maturation antigen (BCMA) (6). Furthermore, all seven CAR-T therapies used second-generation CARs, which contain co-stimulatory protein receptors such as CD28 or 4-1BB (CD137) (7, 8). Both CD28 and 4-1BB can enhance cell proliferation and persistence (9). Emerging data highlights the role of CAR-T cell therapy in targeting CD22, a receptor expressed by mature B cells (10).

However, despite these achievements, relapse rates remain a significant challenge, with studies reporting recurrence in 40–60% of treated patients with hematological malignancies (11). However, developing strategies to improve and optimize the clinical outcomes of CAR-T cell therapy is a major focus of cancer immunotherapy research. The efficiency of T cell therapy is strongly linked to the persistence of these cells and their ability to sustain effector functions over time. These characteristics are central to the concept of T cell fitness (12). T cell fitness refers to the functional capacity of T cells to proliferate, survive, and sustain their cytotoxic activity in response to antigenic stimulation (13). This concept is particularly relevant in the context of CAR-T cell therapy, where the infused T cells must not only target and destroy tumor cells, but also persist long enough to prevent relapse (14). Several factors contribute to T cell fitness, including the phenotypic characteristics of T cells, strength of the signals received during their activation, metabolic state, type of cancer being treated, impact of previous therapies, and timing of T cell collection (15, 16).

This review aims to provide a comprehensive understanding of T cell fitness and its implications for CAR-T cell therapy. Here, we advance the concept that “T cell fitness” represents a higher-order, systems-level property that emerges from the coordinated integration of different mechanistic layers: (i) differentiation state and self-renewal capacity, (ii) signaling architecture and activation dynamics, (iii) metabolic and mitochondrial competence, (iv) epigenetic and transcriptional stability, and (v) host- and treatment-imposed extrinsic pressures. Within this framework, our review aims not only to summarize known determinants of CAR-T performance, but to mechanistically connect them into an integrated model that can be exploited to design fitness-optimized CAR-T products and personalized therapeutic strategies.

## 2 The impact of T cell phenotypes on T cell fitness and their importance in CAR-T cell therapy

T cells are a heterogeneous population of immune cells that exhibit a wide range of phenotypes and functional diversity (17). This diversity is largely determined by the differentiation state of T cells, which can be categorized into several distinct subsets: naïve T cells (TN), central memory T cells (TCM), effector memory T cells (TEM), and Terminally Differentiated Effector Memory RA+ T cells (T EMRA) (17). Each of these subsets plays a different role in the immune response and has varying implications for T cell fitness (18).

TN is the least differentiated subsets (19). These cells are highly proliferative and have the potential to differentiate into other T cell subsets upon encountering their specific antigens (19). TCM expresses markers such as C-C chemokine receptor type 7 (CCR7) and CD62L, which are known for their ability to home in lymphoid tissues and persist for long periods (20). TEM, which lacks CCR7 and CD62L expression, is more differentiated and primarily involved in immediate effector functions (20). T EMRA is the most mature subset and was originally considered ideal for adoptive cell therapies, including CAR-T cell therapy, owing to its potent killing capacity (12). However, previous studies have shown that despite their killing efficacy, T EMRA cells have a poor ability to expand and persist *in vivo* and exhibit low self-renewal ability, reduced homing to tumor niches, and lower survival than memory T cells subpopulations (21, 22). T EMRA is often associated with short-lived immune responses, making it less suitable for CAR-T cell therapy (16). However, TN cells, TCM, and stem cell-like memory T cells (TSCMs) have higher proliferation and persistence potential than effector T cells (23). Therefore, to increase the persistence and enhance the efficacy of CAR-T cells *in vivo*, it is necessary to retain less-differentiated T cells for CAR-T cell therapy (24). In fact, several studies have demonstrated that CAR-T cells from less differentiated subsets such as TN and TCM cells exhibit better persistence and higher proliferative and anti-tumor potentials than products dominated by more differentiated subsets such as TEM and T EMRA cells, which are often prone to exhaustion and rapid attrition (25–29). For instance, recent preclinical studies have demonstrated that CAR-T cells derived from CD4+ and CD8+ TN and TCM subsets display sustained proliferation, greater antitumor potency, and higher persistence *in vivo* than those derived from TEM (30–32).

Moreover, recent research suggests that TSCMs are of particular interest in CAR-T cell therapy because of their enhanced self-renewal capacity and ability to generate all other memory and effector T cell subsets (32, 33). These cells represent a small fraction of the circulating T cell population (approximately 2–3%) but are crucial for maintaining long-term immune surveillance and preventing relapse after CAR-T cell therapy (34). Interestingly, two different clinical trials (NCT02652910 and NCT01087294) have demonstrated that TSCM-derived CAR-T cells possess enhanced metabolic fitness and better expansion capacity than standard CAR-T cells, and mediate robust, long-lasting antitumor activity in patients with recurrent or persistent B-cell malignancies (16).

Overall, these data indicate that early-memory and stem-like phenotypes do not merely correlate with improved CAR-T outcomes, but mechanistically sustain fitness by preserving metabolic plasticity, limiting tonic-signal–induced differentiation, and maintaining epigenetic accessibility of memory-associated loci. Thus, cellular phenotypes constitute the structural foundation upon which other fitness layers are built.

## 3 Enhancing T cell subsets composition to optimize T cell fitness

Given the importance of less-differentiated T cell subsets in determining the efficacy of CAR-T therapy, one of the key strategies to optimize T cell fitness involves enriching these favorable subsets in the final CAR-T product. This can be achieved through selective expansion protocols during the manufacturing process, which enriches for less differentiated T cells while minimizing the presence of highly differentiated, exhausted T cells. Moreover, because cytokines play an important role in the maintenance and expansion of TSCM subsets, several cytokine-targeting strategies have been developed to generate TSCM-like CAR-T cells for adoptive cellular therapy (35).

For instance, a common approach used to improve the efficacy of CAR-T cell therapies is to reduce IL-2 levels, which enriches early memory T cells rather than effector T cells (21). Indeed, CD19-CAR-T cells generated under these conditions provided 10-fold higher cell expansion and displayed potent *in vitro* antileukemic activity (21). Similarly, another study demonstrated that CAR-T cells expanded in IL-15 preserved a less-differentiated TSCM phenotype compared to cells cultured in IL-2 (36). Moreover, the combined use of IL-7 and IL-15 has demonstrated efficient TSCM-like cell expansion and persistence and enhanced the effectiveness of CAR-T cells (37, 38). Additionally, IL-21 has also been shown to be able to promote the generation of TSCM cells (39). Interestingly, adoptive transfer of IL-21-stimulated human CD8+ TSCM efficiently inhibits tumor growth (39). Moreover, IL-10 and IL-21 have been shown to promote the maturation of virus-specific CD8+ T cells (VSTs) into self-renewing TCM (40). These findings pave the way for the design of promising CAR-VST cell products for immunocompromised patients with cancer (41–43).

## 4 Potential role of TCR signal strength and T cell exhaustion on outcomes following CAR-T cell therapy

T cell activation is a complex process that involves (i) the antigen-specific signal presented by major histocompatibility complex (MHC) molecules on the surface of antigen-presenting cells (APCs) through the T cell receptor (TCR), inducing activation of TCR signaling (44) and (ii) the antigen nonspecific signal through co-stimulatory molecules such as CD28 and 4-1BB. Indeed, these co-stimulatory factors play an important role in TCR signaling modulation, and thus improve T cell activation, differentiation, and proliferation upon antigen recognition.

The strength and duration of the TCR signal play a pivotal role in determining the fate of T cells, influencing whether they proliferate and differentiate into effector cells or undergo apoptosis (45, 46). Signal strength is modulated by several factors, including antigen density, the presence or absence of co-stimulatory signals, and the duration of interaction between T cells and APCs (47). A strong and sustained TCR signal, particularly in the presence of co-stimulation, promotes T cell proliferation, differentiation, and the expression of survival factors, such as IL-7 and IL-15 receptors (44). These receptors are crucial for T cell survival, as they enable cells to respond to homeostatic cytokines that prevent apoptosis (44). When TCRs are stimulated, the quantity or quality of the resulting signaling is affected by various factors such as the length and strength of stimulation. Interestingly, differences in the affinities of TCR stimuli can cause differences in T cell physiology (45). Eventually, when naive CD4+ T cells are exposed to extended TCR stimulation, T helper 1 (Th1) cell differentiation is favored over Th2 cell differentiation (48, 49). Conversely, weak TCR signaling favors Th2 cell differentiation (48, 49). Furthermore, weak TCR stimulation is sufficient to enhance memory CD8+ T cell function, while a longer TCR–antigen interaction, the presence of high levels of an antigen, or of a high-affinity antigen are associated with T cell expansion and activation (50, 51).

Complete activation of T cells requires three complementary signals; 1-the interaction of antigenic peptide–MHC complex with TCR, 2- co-stimulatory or co-inhibitory signal provided by antigen-presenting cells, and 3) stimulation by extracellular cytokines, such as IL-2 (52). Building upon these three essential signals for complete T cell activation, early intracellular events are orchestrated by key kinases, among which ZAP-70 serves as a pivotal mediator that bridges TCR engagement to downstream signaling pathways and plays a central role in the early stages of T cell activation. Following TCR engagement, ZAP-70 is recruited to phosphorylated ITAM motifs on the CD3ζ chain, where it is activated and initiates downstream signaling cascades (53). This activation leads to the phosphorylation of key adaptor proteins such as LAT and SLP-76, ultimately triggering calcium flux, MAPK signaling, and transcriptional programs essential for T cell proliferation, differentiation, and effector function (54, 55). ZAP-70 is crucial for the critical link between antigen recognition and full T cell activation, highlighting its indispensable role in T cell immunity (54).

The promotion or inhibition of T cell cytokine production and effector function is determined by the second signal, which decreases inflammation to avoid tissue damage from excessive immune responses, whereas excessive and durative co-inhibitory signals lead to T cell hyporesponsiveness (56). Co-inhibitory signals are mediated by inhibitory receptors expressed on exhausted T cells (57). Indeed, exhausted T cells in the Tumor Microenvironment (TME) are characterized by increased inhibitory receptors and decreased cytokine signaling pathways (58). These inhibitory receptors include programmed cell death protein 1 (PD-1), cytotoxic T-lymphocyte-associated protein 4 (CTLA-4), T cell immunoglobulin and mucin domain 3 (TIM-3), lymphocyte-activation gene 3 (LAG-3), B- and T-lymphocyte attenuator (BTLA), and T cell immunoreceptors with Ig and ITIM domains (TIGIT). Moreover, exhausted T cells show impaired effector cytokine production such as IL-2, tumor necrosis factor-α (TNF-α), interferon gamma (IFN-γ), and Granzyme B (GzmB) (58). T cell exhaustion within the TME renders tumor cells less susceptible to specific TCR-mediated lysis by cytotoxic T cells *in vitro* and increases their tumorigenesis and invasiveness *in vivo*, indicating that the expression of inhibitory markers contributes to immune evasion (58, 59). In summary, T cell exhaustion is a major challenge in CAR-T cell therapy, as it limits the ability of infused T cells to sustain long-term anti-tumor responses.

## 5 T cell exhaustion in hematological malignancies

T cell exhaustion has been identified in several hematological malignancies, including acute myeloid leukemia (AML), chronic lymphocytic leukemia (CLL), ALL, MM, and lymphomas (60–66). For instance, AML patients at risk of relapse post-transplantation had leukemia-specific T cells exhibiting exhaustion markers PD-1, EOMES, and T-bet, which were absent in AML patients who achieved long-term complete remission (CR) (61). Moreover, patients with AML relapse showed a higher proportion of early differentiated TSCM and TCM cells in the bone marrow with multiple inhibitory receptors (61). At relapse, these exhausted T cells exhibit a restricted TCR repertoire, reduced effector functions, and decreased specificity for leukemia antigens (61). Furthermore, in pediatric B-lineage ALL, the interaction mediated by the inhibitory receptor TIM-3 between T cells and leukemia cells is a significant risk factor for relapse, with TIM-3 signaling being the primary mechanism behind T cell dysfunction (63). T cells from patients with CLL exhibit heightened levels of exhaustion markers such as PD-1 (64). These abnormal T cells also display functional deficiencies in proliferation and cytotoxicity, with impaired granzyme packaging and uncoordinated degranulation, contributing to cytolytic defects (64). Furthermore, a subset of exhausted and senescent CD8+ T cells showed downregulation of CD28 and upregulation of CD57 and PD-1, identifying immune dysfunction and predicting relapses in MM following autologous hematopoietic stem cell transplantation (ASCT) (65, 67).

In summary, various types of hematological malignancies display different features of T cell exhaustion as well as relevant cytokines or transcription factors, all associated with abnormalities in immune cells. These observations open new avenues for reversing T cell exhaustion through immunotherapy.

## 6 Optimizing signaling strength and mitigating T cell exhaustion in CAR-T cell therapy for hematological malignancies

Despite its promising success in the treatment of hematological malignancies, CAR-T cell therapy has limitations. The generation of CAR-T cells from exhausted T cells could decrease effector functions, weaken cell proliferation ability, and reduce persistence *in vivo* (68). CAR-T cells from completely responding CLL patients were enriched in memory-related genes, including IL-6/STAT3 signatures, whereas CAR-T cells from non-responders were more differentiated and showed upregulated glycolysis, exhaustion, and apoptosis (69). Moreover, despite the expansion of CAR-T cells *in vivo* and regardless of the presence (or not) of the target antigen on the surface of the tumor cells, relapse occurs after achieving CR in patients with hematological malignancies (70–72). In cases of relapsed/refractory ALL, relapse after CAR-T cell treatment can be separated into two categories: CD19-positive and CD19-negative recurrence (73, 74). CD-19 positive recurrence is related to poor proliferation and killing function of CAR-T cells, which may be due to defective single-chain variable fragment (scFv) binding kinetics and co-stimulatory molecules (75). In CAR-T cell therapy, CARs can elicit different levels of ligand-independent constitutive signaling, designated as tonic signals, and are caused by scFv-mediated CAR self-aggregation (76). Importantly, it has been shown that higher levels of tonic signaling are associated with CAR-T cell-accelerated differentiation and exhaustion, subsequently leading to dysfunction and impaired antitumor effects (76). Therefore, developing strategies to overcome CAR-T cell exhaustion is crucial for enhancing the clinical outcomes of CAR-T cell therapy in hematopoietic malignancies. In contrast, CD19-negative relapse represents bona fide antigen escape under CD19-CAR–imposed selection pressure and is increasingly reported across multiple B-cell malignancies, including ALL, large B-cell lymphoma (DLBCL), and other aggressive non-Hodgkin lymphomas (77–79). CD19-negative leukemic and lymphomatous clones evade CAR-T cell recognition through several distinct but interrelated mechanisms. The most important are structural or expression-level disruptions of CD19, including acquired coding mutations and alternative splicing events, such as exon loss within the FMC63-recognized epitope or aberrant transcripts that mis-localize CD19, which abolishes CAR engagement while preserving malignant fitness (71, 80, 81). Beyond these genetic changes, transcriptional or post-transcriptional repression and defective membrane trafficking can markedly reduce CD19 surface density, for example, through loss of the CD81 chaperone required for proper antigen export (82). A recent study demonstrated that low expression of the transcription factor IKAROS (IKZF1) in pro-B-like B-ALL promotes intron retention within CD19 transcripts, reducing surface antigen expression, and impairing CAR-T recognition (83). Antigen availability can also be depleted through epitope masking, inadvertent CAR transduction that permits cis-CAR–CD19 interaction, and trogocytosis, which transfers CD19 from tumor cells to CAR-T cells (84). Another clinically important mechanism is lineage plasticity or “lineage switch,” particularly in KMT2A-rearranged B-ALL, where leukemic blasts transdifferentiate toward myeloid or early progenitor phenotypes and extinguish B-lineage antigens, giving rise to CD19-null relapse that remains clonally related to the original leukemia (77, 85, 86). These antigen-loss mechanisms are distinct from the T cell intrinsic exhaustion that underlies CD19-positive relapse, highlighting the need for multi-targeting CAR-T Cell Strategies such as CD19/CD22 (5, 87) and vigilant molecular surveillance to pre-empt or counteract CD19 escape in B-cell hematological malignancies.

### 6.1 Transient inhibition of the tonic signaling to prevent CAR-T cell exhaustion

**Figure 1:**
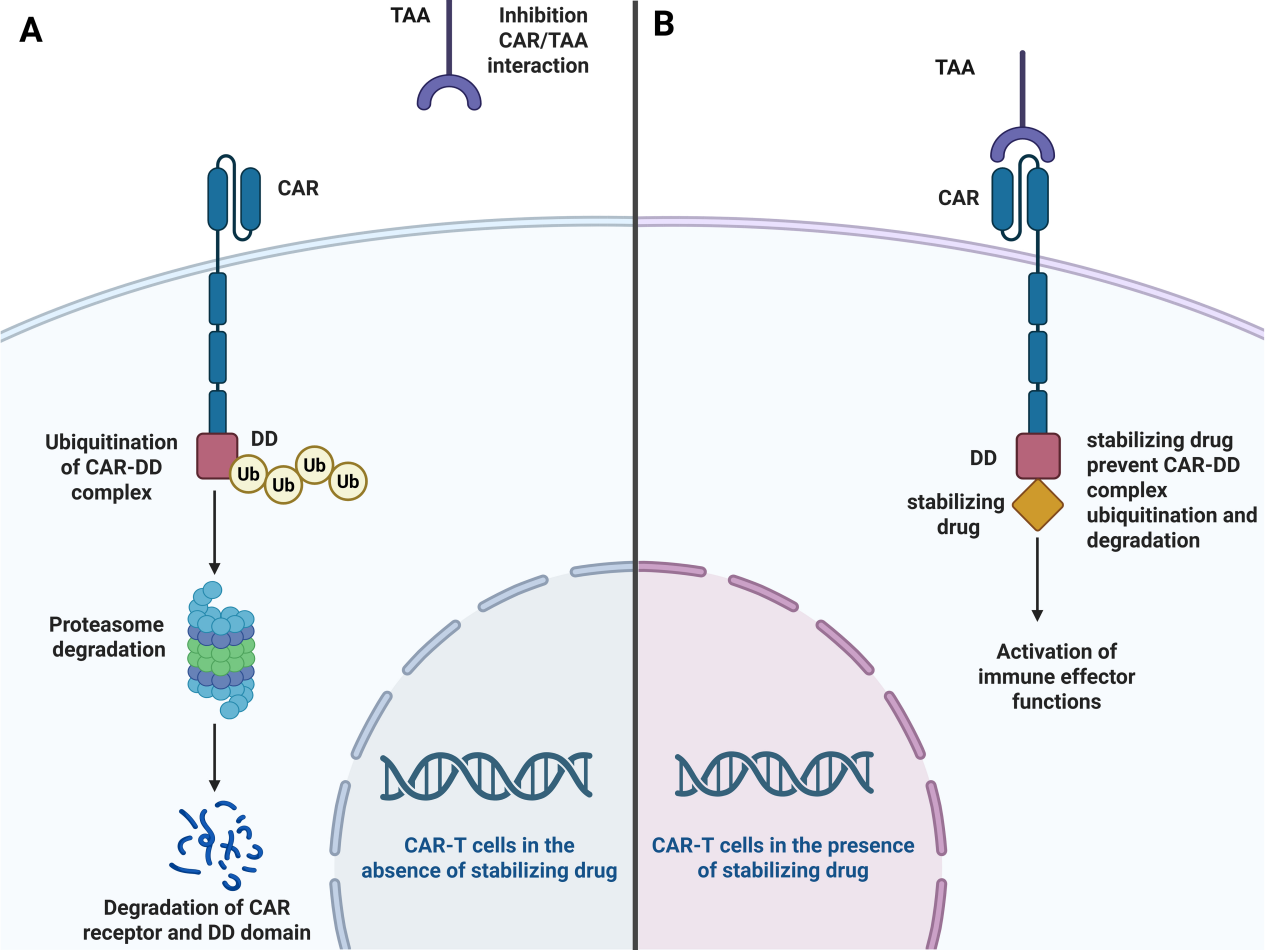
The role of stabilizing drugs in the regulation of CAR-T cell activity. **(A)** In the absence of a stabilizing drug, the destabilizing domain (DD) fused to the CAR leads to ubiquitination of the CAR-DD complex resulting in proteasomal degradation of both the CAR receptor and the DD domain. Consequently, CAR surface expression is decreased, preventing CAR-T cells from recognizing tumor-associated antigens (TAA) and inhibiting immune effector functions. **(B)** When a stabilizing drug is present, it binds to the DD and hinders its ubiquitination, thereby protecting the CAR-DD complex from proteasomal degradation. The intact CAR can then recognize and bind to the TAA on tumor cells, leading to the activation of immune effector functions.

Tonic signaling of CAR, such as spontaneous CAR activation in the absence of tumor antigen stimulation, can promote T cell exhaustion (88). One approach to silencing tonic CAR signaling is to temporarily halt CAR expression when not needed. For instance, there has been a noteworthy report on a system in which a destabilizing domain (DD) was integrated into the CAR construct to enable the drug-dependent control of CAR protein levels (89, 90). In this setup, CAR expression can be terminated by degradation or restored by the administration of a stabilizing agent for DD to prevent CAR degradation (89) (**Figure 1**). This system also serves as a safety measure to manage CAR-T cell-related adverse effects because CAR expression can be controlled by ceasing drug administration (90). Inhibition of the epigenetic modifier EZH2 impairs the reversal of CAR−T cell exhaustion, highlighting its role in shaping the exhaustion-associated epigenetic landscape during tonic CAR signaling. Conversely, the clinically available kinase inhibitor dasatinib can transiently block proximal CAR signaling and effectively rejuvenate exhausted CAR-T cells (89). Therefore, the administration of dasatinib during *ex vivo* expansion of CAR-T cells could represent an additional promising approach for restoring exhausted CAR-T cells without altering the CAR construct. However, while in this preclinical study dasatinib has been shown to modulate proximal CAR signaling and reduce activation/exhaustion programs during production, its effects on *in vivo* toxicity and clinical tolerability may be context dependent. Indeed, a recent report on CD123-CAR-T cells manufactured in the presence of dasatinib (91) for the treatment of pediatric patients with recurrent/refractory leukemia, showed high-grade cytokine release syndrome (CRS) and immune effector cell-associated hemophagocytic syndrome (IEC-HS) and did not demonstrate enhanced clinical benefit compared to expectations from conventional manufacturing protocols. Possible explanations for the heightened inflammatory toxicity observed include differential antigenic density (e.g., CD123 expression on both malignant and healthy hematopoietic cells), CAR design, disease context (AML/myeloid leukemias vs lymphoid malignancies), and host immune milieu. Therefore, the safety profile of dasatinib exposure during CAR-T cell manufacturing requires careful optimization of dosing, timing, and clinical indication, as well as prospective evaluation of safety outcomes across different CAR targets and patient populations.

In addition to self-activation through tonic signaling, various cellular and environmental factors play a significant role in CAR-T cell exhaustion. Several strategies aimed at preventing exhaustion are presented and discussed below:

### 6.2 Blocking of the T cells inhibitory receptors to circumvent T cell exhaustion

**Figure 2:**
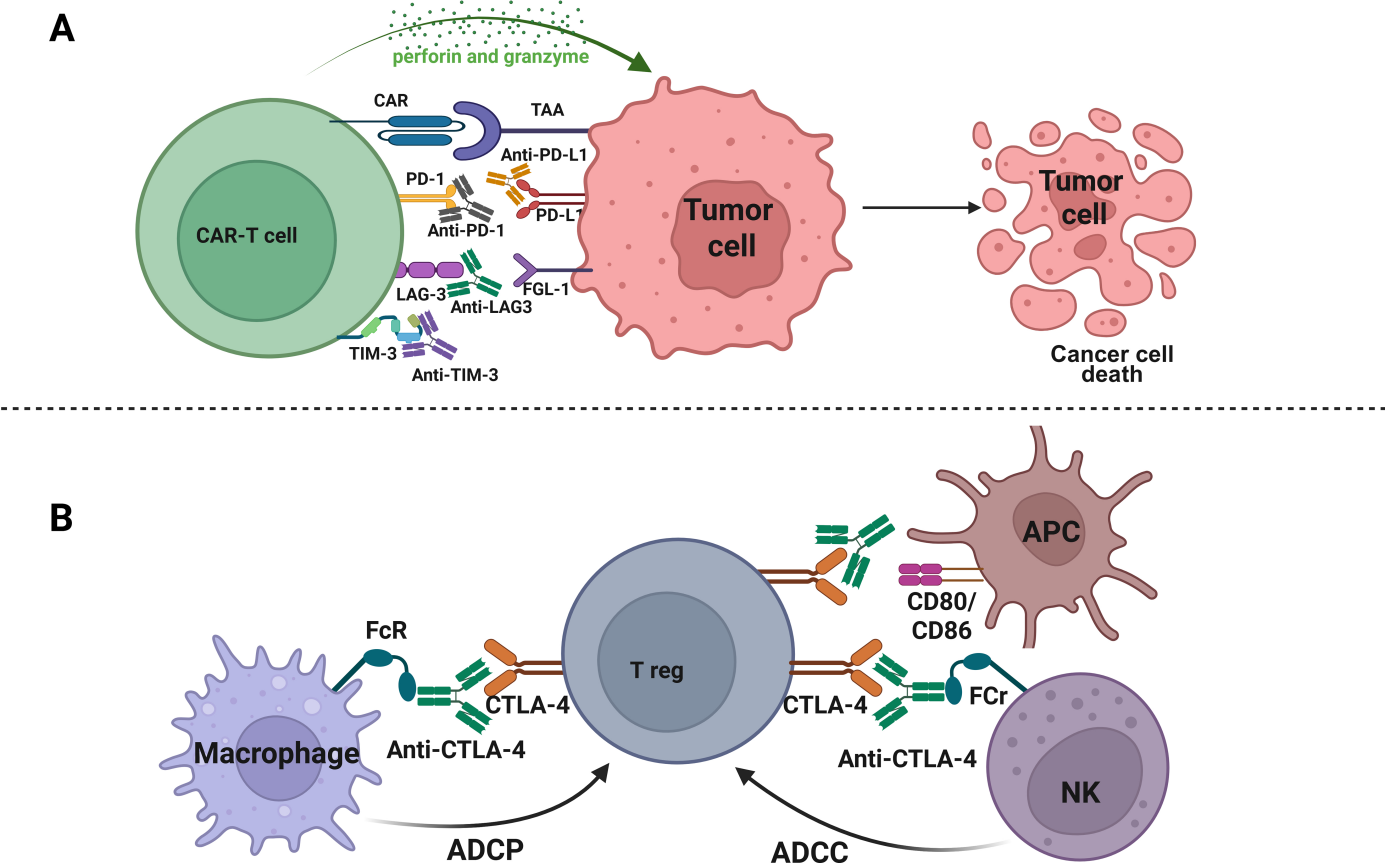
Synergistic enhancement of antitumor immunity through immune checkpoint blockade and Fc−mediated Treg depletion. **(A)** Tumor-associated antigens are recognized by CAR on CAR-T cells, leading to tumor killing via perforin and granzyme release. Co-expression of immune checkpoints such as PD-1, LAG-3, and TIM-3 inhibits T cell anti-tumor activity. Immune checkpoint blockades such anti–PD-1, anti–PD-L1, anti–LAG-3, anti–TIM-3, prevent ligand-mediated inhibition (PD-L1, FGL-1, Galectin-9) and enhance CAR-T cell cytotoxicity against tumor cells. **(B)** Anti–CTLA-4 antibody mediates Treg depletion through innate effector cells. CTLA-4-expressing Tregs are targeted by anti–CTLA-4 antibodies. Through Fc receptor (FcR) engagement, macrophages induce antibody-dependent cellular phagocytosis (ADCP), while NK cells induce antibody-dependent cellular cytotoxicity (ADCC). This depletes tumor infiltrating Tregs and enhances antitumor immune activation via improved CD80/CD86 co-stimulation from antigen-presenting cells (APCs).

**Figure 3:**
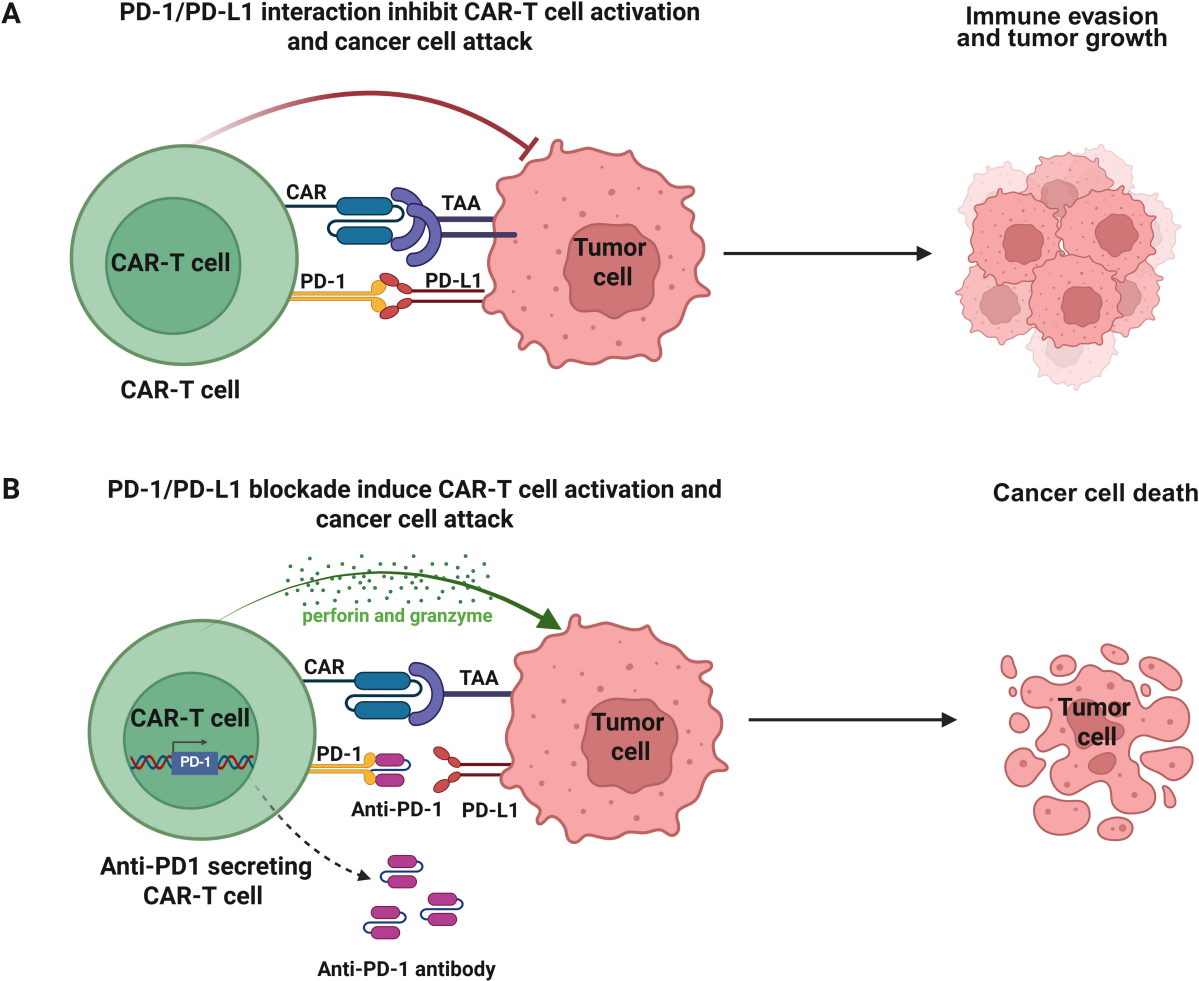
Restoration of CAR-T cell function through PD-1/PD-L1 blockade. **(A)** The interaction of PD-1 on CAR-T cells with its ligand PD-L1 on tumor cells suppresses the CAR-T cell cytotoxicity and cytokine production, leading to immune evasion and tumor growth. **(B)** Engineered CAR-T cells secrete anti-PD-1enhancing their anti-tumor activity. These secreted antibodies block PD-1/PD-L1 interaction, thus improving CAR-T cell function and bystander T cell responses within the tumor microenvironment.

**Figure 4:**
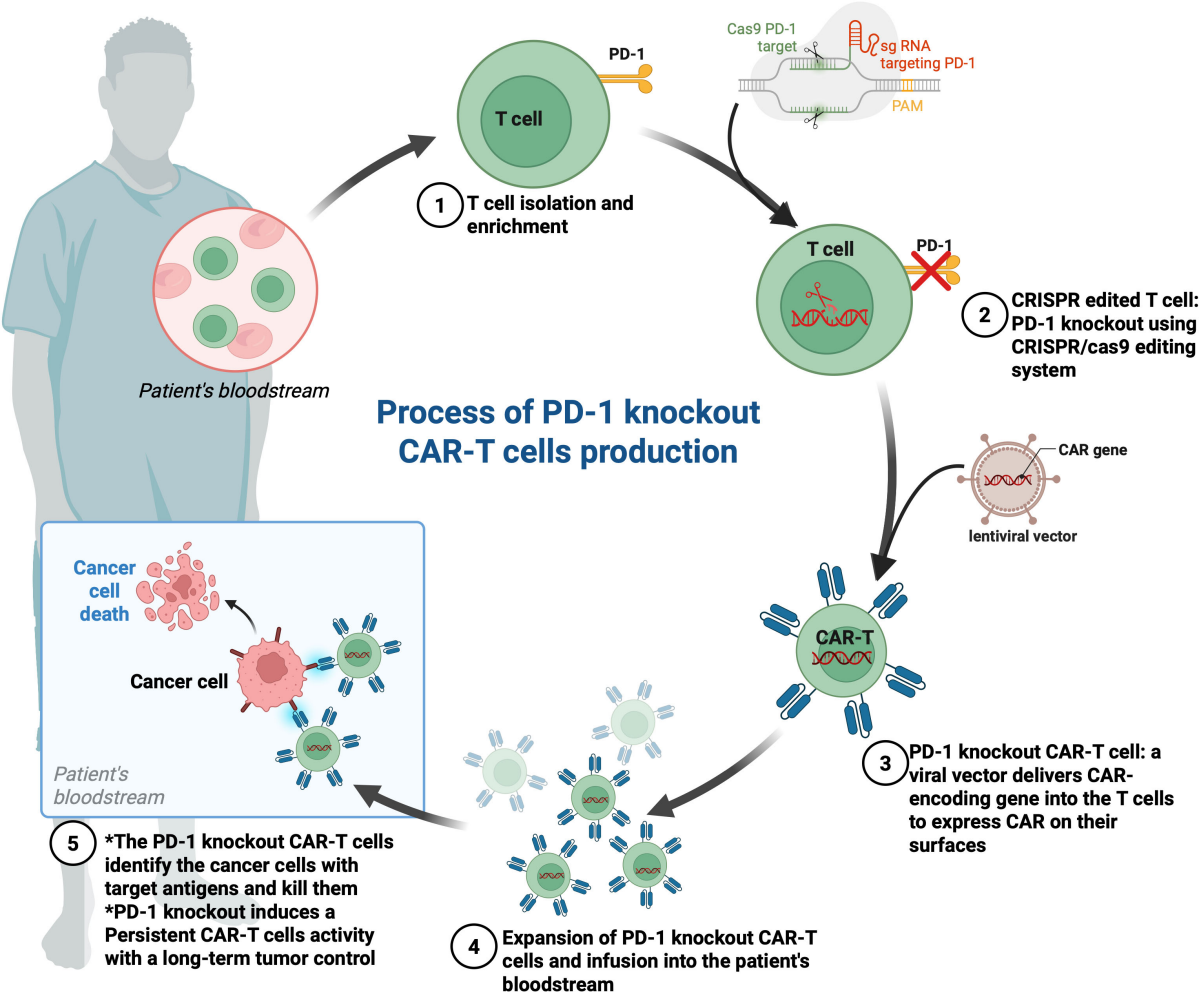
Process of PD-1 knockout CAR-T cells production and function. (1) T cell isolation and enrichment: Peripheral blood is collected from the patient, and T cells are isolated and enriched. (2) PD-1 gene editing: The PD-1 gene is knocked out using a CRISPR/Cas9 system targeting PD-1-specific sequences, preventing PD-1 expression on the T cell surface. (3) CAR transduction: A viral vector (typically lentiviral) delivers the CAR-construct into the T cells, enabling surface expression of a CAR targeting specific tumor antigen. (4) Expansion and infusion: The engineered PD-1 knockout CAR-T cells are expanded ex vivo and reinfused into the patient. (5) Tumor targeting: The infused CAR-T cells identify and kill cancer cells presenting the target antigen. PD-1 deletion enhances CAR-T cell activity and persistence by removing PD-1 inhibitory signaling, resulting in improved long-term tumor control.

Several studies have demonstrated that immune checkpoint blockade is one of the most used and successful strategies to avoid T cell exhaustion (92–94). However, the use of immune checkpoint inhibitors such as anti-PD-1, anti-PD-L1, and anti-CTLA-4 antibodies, independently or in combination with CAR-T cell therapy, has been relatively successful in patients with hematological malignancies (94). Several strategies have been developed to suppress PD-1 function on CAR-T cells, including the combination of anti-PD-1/anti-PD-L1 antibodies (**Figure 2A**), engineering of CAR-T cells secreting anti-PD-1 or anti-PD-L1 antibodies (**Figure 3**), or PD-1 gene silencing (**Figure 4**) (2, 95). Interestingly, CAR-T cells that can secrete anti-PD-L1 or anti-PD-1 antibodies demonstrated higher cytotoxicity and tumor-killing capacity, as well as longer persistence *in vivo* (96, 97). Recently, PD-1 silenced CAR-T cells have been designed and have shown enhanced anti-tumor effects *in vitro* and in different cancer mouse models (95, 98). In this strategy, modifying CAR-T cells using gene silencing technology to block the PD-L1/PD-1 immunosuppression axis mainly involves short hairpin RNA (shRNA) or small interfering RNA (siRNA) gene silencing (99, 100) and CRISPR/Cas9 (clustered regularly interspaced short palindromic repeats/caspase 9) gene-editing technology (101, 102) (**Figure 4**). Interestingly, in a subcutaneous leukemia xenograft, shRNA-mediated gene silencing technology was used to block the effect of PD-1 on the proliferation and anti-tumor effect of CAR-T cells, thereby enhancing its therapeutic effect. In this study, T cells were transduced with the PD-1-shRNA-CAR plasmid using lentivirus to obtain CAR-T cells with PD-1 silenced function (103). The results showed that the efficient PD-1 silencing significantly prolonged the activation and duration of CAR-T cells, resulting in a long tumor-killing effect (103). Building on this strategy of immune checkpoint modulation, anti-CTLA-4 therapy has been shown to enhance T cell priming by disrupting the inhibitory interaction between CD80/86 and CTLA-4, as well as depleting regulatory T cells (Tregs) through antibody-dependent cellular cytotoxicity (ADCC) and phagocytosis (ADCP) (104) (**Figure 2B**). To further potentiate CAR-T cell function, additional checkpoints, such as CTLA-4, TIM-3, and LAG-3, have also been targeted using gene editing technologies, thereby overcoming multiple layers of immune suppression and enhancing anti-tumor efficacy (105). Interestingly, a double knockout of PD-1 and CTLA-4 using the CRISPR/Cas9 system effectively improved the anti-tumor effects of CAR-T cells (98).

These preclinical data demonstrate that the blockade of T cell inhibitory receptors is a key factor in enhancing the therapeutic effects of CAR-T cells.

### 6.3 Inhibition of immunosuppressive cells and cytokines associated with T Cell exhaustion

**Figure 5:**
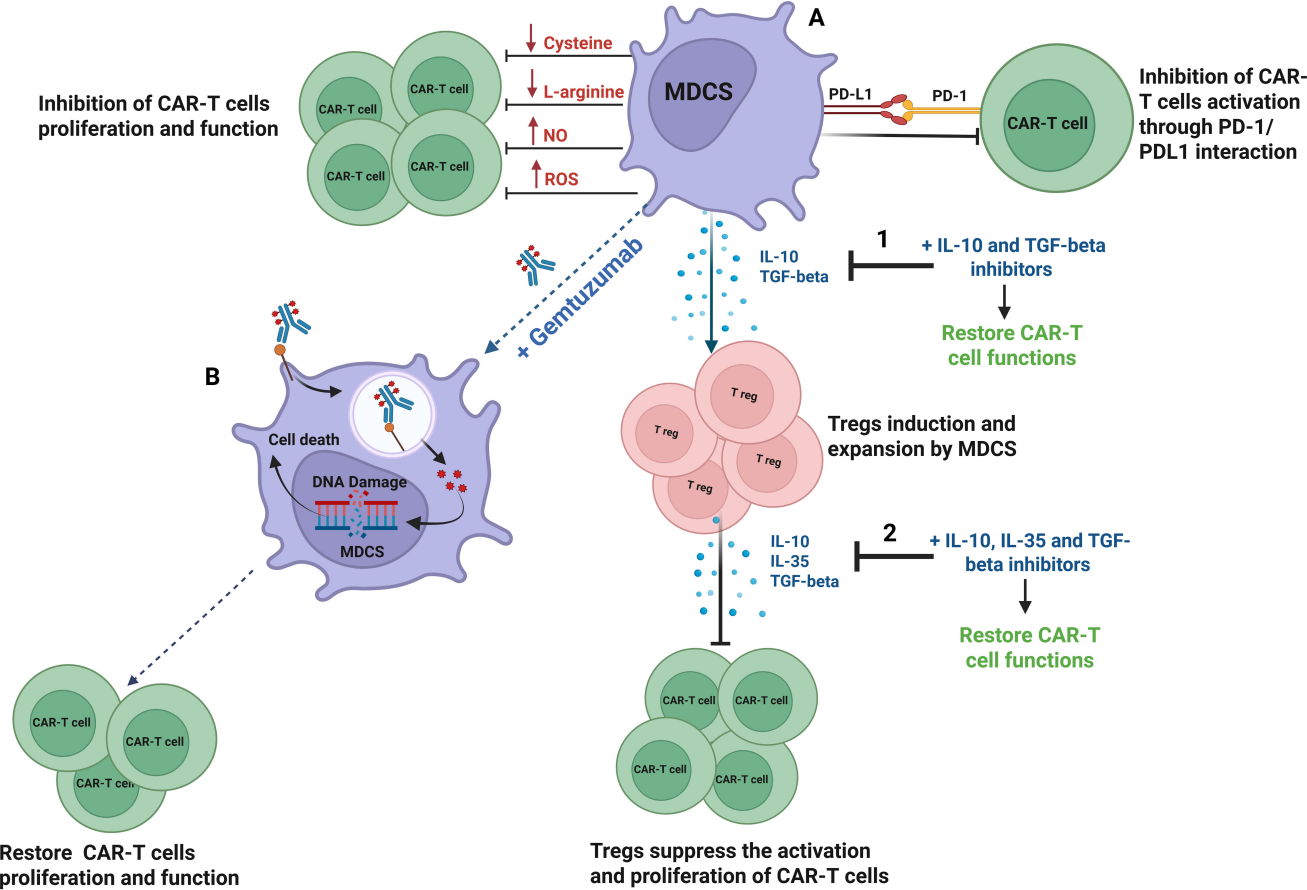
Blocking MDSCs with Gemtuzumab/Ozogamicin or cytokine inhibitors restores CAR-T cell function. **(A)** Myeloid-derived suppressor cells (MDSCs) impair CAR-T cell activity by expressing PD-L1 to induce exhaustion, promoting regulatory T cell expansion via IL-10, IL-35 and TGF-β, and disrupting metabolism through nutrient depletion and the release of nitric oxide (NO) and reactive oxygen species (ROS). **(B)** Treatment with gemtuzumab, an anti-CD33 antibody-drug conjugate, selectively targets and eliminates MDSCs by inducing DNA damage. MDSCs death promotes CAR-T cell proliferation and activation. In addition, the use of inhibitors or antibodies targeting IL-10, IL-35, and TGF-β signaling can disrupt the suppressive cytokine environment maintained by MDSCs (1) and Tregs (2) and restore CAR-T cells proliferation and function.

Myeloid-derived suppressor cells (MDSCs) represent a critical component of the immunosuppressive tumor microenvironment and pose a significant barrier to CAR-T cell therapy efficacy (106). MDSCs impaired CAR-T cell proliferation and cytotoxic function through several interconnected mechanisms (**Figure 5A**). One major pathway involves immune checkpoint signaling, wherein MDSCs express PD-L1, which binds to PD-1 on CAR-T cells, inducing T cell exhaustion and diminishing activation (107). In parallel, MDSCs promote the expansion of Tregs, a subset of immunosuppressive lymphocytes that inhibits effector T cell function and proliferation. This is mediated through the secretion of cytokines such as IL-10 and TGF-β (108). These Tregs further suppress CAR-T cell activity by releasing IL-10, IL-35, and TGF-β (109). Additionally, MDSCs contribute to metabolic suppression by depleting essential nutrients, such as cysteine and l-arginine, and producing inhibitory metabolites, such as nitric oxide (NO) and reactive oxygen species (ROS), collectively impairing CAR-T cell viability and adversely affecting persistence (110).

To overcome these immunosuppressive constraints, several strategies aimed at inhibiting Treg and MDSC activity, in conjunction with CAR-T cell therapy, have demonstrated significantly enhanced antitumor responses in various preclinical cancer models. In fact, hampering Treg activity in combination with CAR-T cell therapy has been shown to significantly enhance antitumor responses in various preclinical models of hematologic and solid malignancies (111). Building on this, Long et al. demonstrated that combining GD2-targeted CAR-T cells with strategies to neutralize the immunosuppressive effects of myeloid-derived suppressor cells (MDSCs) markedly improved CAR-T cell efficacy in a sarcoma xenograft model (112). Based on these findings, pharmacological approaches, such as the use of immunotoxins, such as gemtuzumab ozogamicin, have proven effective in depleting MDSCs, thereby further augmenting CAR-T cell responses across multiple tumor types (113) (**Figure 5B**). Hence, targeting these immunosuppressive cells presents an additional promising avenue to enhance the efficacy of CAR-T cell therapy. Moreover, inhibition of immunosuppressive factors such as IL-10, IL-35, and TGF-β can lead to significant upregulation of T cell memory-related genes and downregulation of exhausted genes (114) [**Figures 5A, B**]. For example, lenalidomide has been shown to reverse the T cell exhaustion by inhibiting IL-10-induced STAT3 (signal transducer and activator of transcription 3) phosphorylation in patients with CLL (115). A preclinical study demonstrated that silencing endogenous TGF-β receptor II (TGFBR2) in CAR-T cells using CRISPR/Cas9 technology could reduce Treg conversion, prevent CAR-T cell exhaustion, and enhance tumor-killing efficacy in xenograft cancer models (116).

### 6.4 Inhibition and tuning of transcriptional regulators of T cell activation and exhaustion programs

Exhaustion and durable persistence in chronically stimulated T cells (including tumor-infiltrating CAR-T cells) are controlled by interconnected transcriptional circuits that govern lineage progression, effector competence, and epigenetic stability. In this context, TOX/TOX2 and NR4A family members are widely implicated in sustaining exhaustion phenotypes (117, 118). Accordingly, genetic disruption of the TOX/NR4A axis has been shown to reduce dysfunction and improve antitumor activity of CAR-T cells in solid tumor models, including enhanced tumor control with TOX/TOX2 double deficiency and improved efficacy with NR4A ablation (119–124). Recently, it has been shown that the hematopoietic progenitor kinase 1 (HPK1) -NFκB-Blimp1 axis mediates T cell dysfunction, and that high expression of MAP4K1 (which encodes HPK1) correlates with worse patient survival in several cancer types (125). This study suggests that HPK1 is an attractive target for improving the response to CAR-T cell therapy (125). Moreover, overexpression of the transcription factors BATF and IRF4 cooperates to counter exhaustion in tumor-infiltrating CAR-T cells (126). In contrast, TCF1 has been established as a hallmark and functional regulator of progenitor/stem-like exhausted CD8 T cells that retain proliferative potential, sustain a self-renewing pool, and can give rise to more terminally exhausted state. This feature underlies durable responses and responsiveness to immunomodulatory strategies in chronic contexts (127, 128). Consistent with this model, pathways that preserve or restore TCF1-linked programs can enhance persistence: for example, Regnase−1 has been reported to restrain TCF1-associated memory/stem-like features, and its disruption can augment TCF1^+^ CAR-T persistence and antitumor function in relevant settings (117). In ALL, Regnase−1 knockout enhanced TCF1^+^ CAR−T cell persistence and antitumor activity (129). Accordingly, strategies that preserve or enhance TCF1-associated programs can support long-term persistence and functional plasticity of CAR-T cells, especially under chronic antigen exposure.

Similarly, BACH2 functions as a lineage/fate regulator, promoting memory-type programs and mitigating tonic signal–induced dysfunction during CAR-T cell manufacturing (130, 131). However, sustained or dysregulated BACH2 expression may constrain effector differentiation, implying that dynamic tuning rather than constitutive gain or loss may be required for optimal long-term efficacy across CAR constructs and disease contexts. Additional transcriptional strategies that enhance functional resilience include reinforcing AP-1 network balance (e.g. c-Jun) which has been shown to enhance expansion potential, increase functional capacity, diminish terminal differentiation, and improve anti-tumor potency in multiple different preclinical models (132).

These findings highlight that effective manipulation of transcriptional programs in CAR-T cells requires a nuanced understanding of developmental hierarchies and context-dependent function: some factors (e.g., TOX/NR4A) contribute to dysfunctional programs that can be selectively targeted, while others (e.g., TCF1, BACH2) play dual roles in maintaining progenitor potential and influencing effector fate.

Beyond transcription factors, T cell functions are also modulated by metabolic changes. The next section explores metabolic reprogramming and its impact on T cell function and fitness.

## 7 T Cell metabolism: metabolic reprogramming in T cell activation & fitness

T cell metabolism is an intricate dynamic process linked to the functional state of T cells. Different T cell subsets have specific functions in the immune system. Therefore, they possess distinct metabolic pathways. However, quiescent naïve T cells rely on oxidative phosphorylation (OXPHOS) and fatty acid oxidation (FAO) to meet their energy requirements (133). This metabolic pathway is efficient in generating ATP but does not support the high biosynthetic demands of proliferating cells. Upon activation, T cells undergo a metabolic switch to glycolysis and glutaminolysis, which provides rapid ATP production, synthesis of nucleotides, amino acids, and lipids necessary for rapid proliferation of effector functions of the cells, and cytotoxicity (134). This metabolic shift is driven by multiple signaling pathways and transcription factors, particularly the *MYC* oncogene, which plays a central role in regulating metabolic reprogramming during T cell activation (134). Additionally, fatty acid oxidation has been implicated in the survival and function of memory T cells, highlighting the importance of diverse metabolic pathways in maintaining T cell fitness (135). In fact, from their nascent stages and throughout development, T cells transition between metabolic quiescence and activation (136). Metabolic reprogramming is governed by key receptor signaling events, growth factor cytokines, and nutrient availability (137).

Interestingly, the TME poses unique metabolic challenges that can impair T cell function. Tumor-infiltrating T cells often exhibit a dysfunctional metabolic state, characterized by reduced glycolytic capacity and increased oxidative stress, which can lead to T cell exhaustion (138). This metabolic dysfunction is associated with the upregulation of immune checkpoint molecules, such as PD-1 and CTLA-4, which further suppress T cell metabolism and function (134, 138). Strategies to enhance T cell metabolic fitness in the TME, such as targeting metabolic pathways or modulating the expression of key metabolic regulators, are being explored as potential therapeutic approaches to improve anti-tumor immunity (134, 138). Tregs exhibit distinct metabolic adaptations that support their survival and function in the TME. For instance, CD36-mediated lipid metabolism has been shown to enhance the metabolic flexibility of Tregs, allowing them to adapt to the nutrient-poor conditions often found in tumors (139). Moreover, the role of autophagy in regulating Treg metabolism and stability has been highlighted, with disruptions in autophagic processes leading to metabolic dysregulation and impaired Treg function (140, 141).

Likewise, the co-stimulatory molecule CD28 on T cells promotes glycolytic metabolism, which improves T cell activation, proliferation, and persistence and thus optimizes CAR-T therapies in cancer patients (142).

Overall, T cell metabolism and metabolic reprogramming are integral to T cell activation, differentiation, and fitness. Moreover, the dynamic nature of T cell metabolism, influenced by various intrinsic and extrinsic factors, underscores the complexity of T cell biology and its implications for immunotherapy and disease management (143).

### 7.1 The role of mitochondrial fitness in T cell function

The interplay between T cell metabolism and epigenetic regulation is a major area of active research. Metabolic changes can influence the epigenetic landscape of T cells, thereby affecting gene expression and functional outcomes (144). Indeed, mitochondrial membrane potential has been linked to the stemness and longevity of T cells, suggesting that metabolic states can have lasting effects on T cell differentiation and memory (145). In addition, mitochondrial dynamics, including fission and fusion, are essential for maintaining mitochondrial quality control and adapting to changing cellular conditions. Studies have shown that mitochondrial dynamics influence T cell differentiation into effector and memory subsets. For example, effector T cells responsible for immediate pathogen clearance exhibit fragmented mitochondria, favoring glycolysis as the primary energy source (146). Conversely, memory T cells, which provide long-term immunity, display elongated mitochondria and rely on OXPHOS (147). Mitochondrial dysfunction, marked by reduced OXPHOS, increased reactive oxygen species (ROS) production, and altered mitochondrial dynamics, has been implicated in T cell exhaustion (148). In addition, accumulation of damaged mitochondria and impaired mitophagy contributes to a decline in mitochondrial fitness (149).

**Figure 6:**
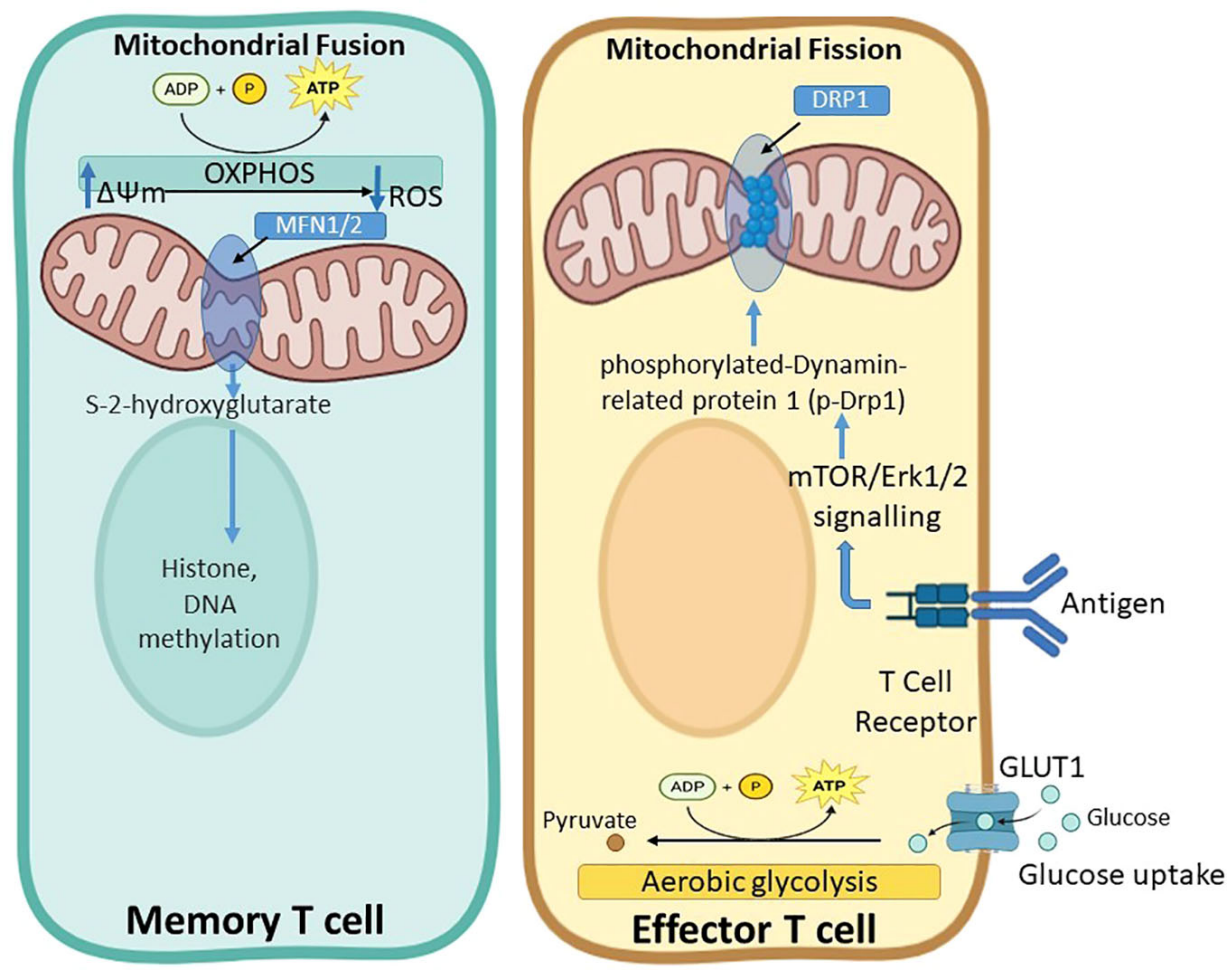
Mitochondrial dynamics regulate metabolic programming and function in memory and effector T cells. Mitochondrial fusion, driven by mitofusin proteins MFN1/2, is associated with memory T cells and supports oxidative phosphorylation (OXPHOS), increased mitochondrial membrane potential (ΔΨm), reactive oxygen species (ROS) production, and S-2-hydroxyglutarate generation, which influences histone and DNA methylation to promote long-term survival and persistence. In contrast, mitochondrial fission, regulated by the phosphorylation of Dynamin-related protein 1 (p-Drp1) downstream of mTOR/Erk1/2 signaling, predominates in effector T cells. Upon T cell receptor engagement and antigen stimulation, effector T cells upregulate GLUT1-mediated glucose uptake and shift toward aerobic glycolysis, resulting in increased pyruvate metabolism and rapid ATP generation to support proliferation and cytotoxic function.

Mitochondrial fitness, reflected by parameters such as spare respiratory capacity (SRC) and mitochondrial membrane potential (ΔΨm), is critical for sustaining T cell activity under stress conditions, such as during an immune response (150). A high SRC provides a reserve capacity that allows T cells to meet increased energy demands during periods of heightened activity, whereas a high ΔΨm is associated with increased expression of exhaustion markers and higher levels of ROS (145, 150) (**Figure 6**).

In CAR-T therapy, mitochondrial fitness plays a crucial role in determining the long-term persistence and efficacy of infused T cells (151). Impaired mitochondrial function, characterized by low SRC and increased ROS production, has been linked to T cell exhaustion and reduced therapeutic efficacy (152). This is particularly relevant in diseases such as chronic lymphocytic leukemia (CLL), in which prolonged exposure to tumor cells can impair mitochondrial function, leading to a reduced ability of T cells to mount an effective anti-tumor response (152). In addition, the co-stimulatory molecule 4-1BB promotes mitochondrial fitness and memory-like differentiation (142).

Mitochondrial fitness is pivotal for next-generation immunotherapies aimed at restoring T cell functionality. Targeting mitochondrial biogenesis and ROS regulation may enhance T cell differentiation, activation, persistence, and resistance to exhaustion, offering therapeutic benefits in chronic infections and cancer (153).

### 7.2 Metabolic interventions to enhance T cell fitness in CAR-T cell therapy

Metabolic interventions can improve T cell fitness and persistence of CAR-T cells, thereby enhancing their efficacy in hematological cancer treatment (154). Metabolic limitations may significantly impair T cell physiology, particularly in the context of CAR-T cell therapies (15). Therefore, strategies aimed at enhancing T cell metabolism are critical for improving the efficacy of CAR-T therapies and their anti-tumor activity.

One promising approach involves modulation of signaling pathways that regulate T cell metabolism. However, overexpression of FOXO1 has been shown to enhance CAR-T cell polyfunctionality and metabolic fitness, leading to improved antitumor efficacy without significant toxicity (155). Similarly, co-stimulatory CD28 has been shown to improve T cell glycolytic metabolism, while receptor 4-1BB has been demonstrated to induce mitochondrial biogenesis and enhance metabolic function in T cells, thereby supporting their survival and activity in the TME (156). Thus, engineered CAR-T cells expressing either CD28 or 4-1BB co-stimulatory domains show high efficacy in patients with relapsed hematological malignancies (142).

Additionally, manipulation of cytokine signaling has been explored to enhance CAR-T cell metabolism. Transgenic expression of IL-7 regulates CAR-T cell metabolism and improves persistence *in vivo* (157). In addition, IL-15 preconditioning has been reported to augment CAR-T cell responses, promoting a stem-like phenotype associated with enhanced engraftment and antitumor activity (158). Interestingly, IL-10-expressing CAR-T cells have shown less T cell exhaustion in the TME through sustained mitochondrial fitness and increased OXPHOS, resulting in the enhanced proliferative capacity and effector function of CAR-T cells (159).

Moreover, the reprogramming of lipid metabolism has emerged as a novel strategy to prevent T cell senescence and enhance therapeutic efficacy. An interesting report demonstrated that inhibiting Cytosolic phospholipase A_2_ (cPLA2α) can reprogram lipid metabolism in T cells, resulting in improved antitumor immunity (160). Moreover, it has been reported that a low-arginine microenvironment impairs CAR-T cell proliferation, limiting its efficacy in clinical trials against hematological and solid malignancies (161, 162). To overcome this limitation, modified CAR-T cells expressing argininosuccinate synthase (ASS) and ornithine transcarbamylase (OTC) have been designed to increase proliferation and promote tumor clearance without influencing CAR-T cell cytotoxicity (162). Moreover, CAR-T cells with high glycolytic metabolism and low terminal differentiation demonstrated better CAR-T product outcomes (69). By enhancing the metabolic fitness of T cells, these interventions can potentially overcome the suppressive effects of the TME and boost CAR-T cell efficacy. Moreover, strategies such as increasing mitochondrial spare respiratory capacity (SRC) and reducing ROS production are being explored for their potential to enhance CAR-T cell performance (163). These approaches may involve the use of drugs that promote mitochondrial biogenesis as well as inhibitors targeting tumor-specific metabolic pathways, reducing competition for energy resources between tumor cells and CAR-T cells (164). In addition, activation of peroxisome proliferator-activated receptor gamma coactivator 1-alpha (PGC-1α), a key regulator of mitochondrial biogenesis, has been shown to improve the mitochondrial fitness of T cells and enhance their anti-tumor activity (165, 166).

Metabolic and mitochondrial fitness act as the energetic backbone of T cell fitness, enabling sustained signaling, resistance to exhaustion-associated transcriptional programs, and survival within nutrient-restricted tumor microenvironments.

In summary, leveraging metabolic reprogramming offers a promising approach for enhancing T cell fitness in CAR-T cell therapy (**Table 1**). Future research should focus on integrating these metabolic strategies into clinical protocols to maximize the therapeutic potential of CAR-T therapies.

**Table 1:** Overview of metabolic interventions and their roles in enhancing T cell fitness for improved efficacy in CAR-T cell therapy.

Type of metabolic intervention	Role in enhancing T cell fitness in CAR-T cell therapy	References
Pathway Modulation	Overexpression of FOXO1 enhances polyfunctionality and metabolic fitness, improving antitumor efficacy without toxicity.	(155)
	Activation of the costimulatory receptor 4-1BB induces mitochondrial biogenesis, supporting survival and activity in the TME.	(156)
	Inhibition of MEK1/2 signaling maintains T cell activation and proliferation	(167)
	Inhibition of PI3K prolong the survival of CAR-T cells and increase their effectiveness in killing tumor cells in vivo by keeping the cells less differentiated in vitro without affecting their activation	(168) (169)
Cytokine Signaling	Transgenic IL-7 expression regulates CAR-T cell metabolism and improves in vivo persistence.	(157)
	IL-15 preconditioning promotes a stem-like phenotype, enhancing engraftment and antitumor activity.	(158)
	IL-10-expressing CAR-T cells prevent T cells exhaustion in the TME through sustained mitochondrial fitness and increased OXPHOS in an MPC-dependent manner, enhancing theproliferative capacity and effector function of CAR-T cells	(159)
Metabolism Reprogramming	Inhibition of cPLA2α reprograms lipid metabolism, preventing T cell senescence and improving antitumor immunity.	(160)
	Mitigates metabolic competition between T cells and tumor cells, enhancing T cell function.	(10)
	Designing of ASS or OTC-modified CAR-T cells to increase proliferation and promote tumor clearance without influencing the cytotoxicity or exhaustion of CAR-T cells.	(162)
	Production of CAR-T cells with high glycolytic metabolism and low terminal differentiation for better outcomes of CAR-T products.	(69)
Mitochondrial Fitness Enhancement	Enhancing mitochondrial fitness by increasing SRC and reducing ROS production boosts CAR-T cell performance.	(163)
	Activation of PGC-1α improves mitochondrial biogenesis and anti-tumor activity of T cells. PGC1α agonist, with anti-PD-1 treatment resulted in increased OXPHOS and decreased T-cell apoptosis	(165) (170)
	Inhibition of the mitochondrial pyruvate carrier (MPC) aid in the differentiation of memory T cells and increases CAR-T cell anti-tumor effects.	(171)
	Use of drugs promoting mitochondrial biogenesis and inhibitors targeting tumor-specific metabolic pathways reduces energy competition in the TME.	(164)
	Inhibition of β2-AR signaling increases mitochondrial biogenesis, respiration capacity, and membrane potential	(172)
	Blocking IDH2 activity by gene knockdown or targeted drugs can remodel the central carbon metabolism pattern and epigenetic regulation of CAR T cells and increase the anti-tumor ability of CAR T cells.	(173)

## 8 CAR architecture as a determinant of T cell fitness: Linking co-stimulation, tonic signaling, and antigen-binding properties to functional and metabolic outcomes

The intrinsic structural design of the chimeric antigen receptor constitutes a major determinant of T cell fitness. CAR architecture governs the magnitude, duration, and qualitative nature of intracellular signaling, thereby shaping differentiation trajectories, metabolic programming, susceptibility to exhaustion, and long-term persistence (174). Key design parameters include the choice of co-stimulatory domain, the affinity and structural properties of the single-chain variable fragment (scFv), CAR surface density, and the propensity for ligand-independent receptor clustering and tonic signaling (88, 175, 176).

Among these features, the intracellular co-stimulatory domain represents one of the most influential modulators of CAR-T cell biology. Importantly, CAR-induced signaling architecture and metabolic programming are tightly interconnected. Strong CD28-driven signaling favors aerobic glycolysis, increased glucose uptake, and rapid biosynthetic activity, whereas 4-1BB–based constructs enhance mitochondrial mass, oxidative metabolism, and resistance to oxidative stress (175, 177). In line with these observations, a recent study in patients with relapsed/refractory diffuse large B-cell lymphoma (DLBCL) demonstrated that CAR-T cells from responders exhibited a balanced metabolic program integrating both glycolytic and mitochondrial capacity, whereas non-responders displayed polarized metabolic states, characterized by excessive glycolysis in CD28-based CAR-T cells or suppressed glycolysis in 4-1BB-based constructs (177). These findings highlight co-stimulatory domain selection directly links CAR structure to metabolic trajectories and exhaustion vulnerability, positioning CAR design as a determinant of therapeutic outcome and a potential avenue for rational personalization of CAR-T products.

On the other hand, the antigen-binding domain further modulates signaling strength and cellular fate. High-affinity scFvs and elevated CAR surface expression increase the probability of sustained receptor engagement, ZAP-70 recruitment and amplifying downstream signaling cascades (MAPK, NFAT, and NF-κB signaling cascades) (176, 178). In addition, excessive or prolonged activation promotes sustained calcium influx, transcriptional activation of exhaustion-associated regulators (TOX, NR4A), and over-metabolic glycolysis, accelerating terminal differentiation and functional collapse (118, 179). Conversely, moderate-affinity scFvs and controlled CAR surface density can preserve antigen sensitivity while limiting chronic signaling burden, maintaining metabolic flexibility and delaying exhaustion (180). Tonic signaling represents a particularly important structural consequence of CAR design. Even in the absence of antigen, CAR-induced constitutive signaling can be due to scFv-mediated receptor self-aggregation and specific hinge or transmembrane configurations (88). Such ligand-independent activation accelerates differentiation, reshapes chromatin accessibility, and promotes stable exhaustion-associated programs, ultimately reducing proliferative capacity and antitumor efficacy. These effects highlight that exhaustion is not merely a function of tumor burden or chronic antigen exposure but can occur during manufacturing through CAR-intrinsic signaling properties.

Collectively, these data redefine CAR architecture as a programmable therapeutic parameter rather than a passive antigen-recognition scaffold. Rational selection and optimization of co-stimulatory domains, scFv affinity, receptor density, and tonic signaling behavior enable direct modulation of T cell metabolic resilience, differentiation speed, and exhaustion susceptibility.

Integrating receptor-level engineering with cytokine conditioning, metabolic interventions, and transcriptional circuit modulation provides a framework for constructing next-generation CAR-T products with enhanced durability and broader efficacy across heterogeneous disease contexts. Building on this framework, the following section examines how patient-specific biological and clinical factors further shape T cell fitness and influence therapeutic outcomes.

## 9 The impact of patient-specific factors on T cell fitness

### 9.1 The interplay of chronic inflammation and T cell fitness

Optimal T cell fitness ensures a rapid and robust immune response to infections, cancer, and other immune challenges, and plays a vital role in overall immune health and disease defense. In contrast, chronic inflammation may be perceived as a state of prolonged and persistent inflammatory response that occurs when the immune system continues to attack perceived threats long after the initial injury or infection has resolved (181). Interestingly, chronic inflammation and T cell fitness are closely interconnected, with each influencing the other (182). Chronic inflammation can lead to the continuous activation of T cells, gradually impairing their fitness, affecting their function, promoting tumor development, and suppressing anti-tumor immunity. In addition, T cells with memory-like phenotypes are known to retain higher fitness, underscoring the impact of the host inflammatory environment on T cell quality and therapeutic potential (183). Indeed, prolonged exposure to inflammatory signals can cause T cell exhaustion and consequently limit their ability to effectively control infections and tumors (184). For example, in chronic inflammatory settings such as CLL, memory-like T cells with lower exhaustion exhibit superior function, whereas exhausted, inflammation-driven T cells with metabolic dysregulation display reduced fitness and are associated with poorer responses (69, 185). Further, chronic inflammation can contribute to a decline in the diversity of the naive T cell repertoire, limiting the immune system’s ability to recognize and respond to novel antigens (186). This decline is particularly pronounced with aging, and can increase susceptibility to infections and reduce vaccine efficacy (187). In particular, senescence-associated T cells (SA-T cells) secrete proinflammatory factors that contribute to chronic inflammation and autoimmunity (188). In this context, inflammatory mediators are known to accelerate T cell senescence, causing T cells to lose their functional capacity and contributing to the overall inflammatory milieu (189). Several inflammatory mediators play key roles in modulating T cell fitness during chronic inflammation. Indeed, pro-inflammatory cytokines such as tumor necrosis factor alpha (TNF-α) and IL-6 have been shown to alter T cell signaling thresholds, leading to impaired activation and function, particularly in the context of aging (184) (189). Furthermore, eicosanoids, which are inflammatory lipids such as prostaglandins, can promote inflammation and affect T cell function (190).

(190). Therefore, understanding the mechanisms by which inflammation affects T cells is crucial to develop strategies to mitigate these effects and improve immune health.

### 9.2 Impact of aging on CAR-T cell fitness

Aging contributes to immunosenescence, marked by a decline in naïve T cell production and diminished T cell diversity (191). As T cells age, they exhibit reduced proliferative capacity, impaired cytokine production, and increased expression of inhibitory receptors, all of which contribute to T cell exhaustion and decrease the effectiveness of CAR-T therapy in older patients (191). Furthermore, aging significantly compromises T cell fitness, a decline shaped by various patient-specific factors. Therefore, in older patients, CAR-T cell therapy efficacy can be compromised by poor fitness of the starting T cell material (192).

Mitochondrial dysfunction is another consequence of aging that further impairs the fitness and functionality of T cells, exacerbating the challenges in achieving optimal CAR-T cell efficacy in elderly individuals (192).

T cell senescence leads to loss of proliferative capacity and increased accumulation of dysfunctional CD4+ T cells, termed senescence-associated T (SA-T) cells (188).

Thus, efficient metabolic programming is crucial for T cell homoeostasis and differentiation (193). Age-related metabolic dysregulation can impair T cell responses, particularly in the context of infections and vaccinations (193). Moreover, genome-wide analyses have revealed significant transcriptional changes in aged T cells, affecting gene expression and signaling pathways crucial for T cell function (194).

While these factors highlight the challenges of T cell aging, ongoing research aims to develop interventions that could rejuvenate T cell function, potentially improving the outcomes of immunotherapies such as CAR-T cell therapy for older patients (192).

### 9.3 Impact of hematological cancer type on CAR-T cell fitness

The type of cancer being treated significantly influences the fitness of T cells used in CAR-T therapy (195). Different cancers have distinct tumor microenvironments that can either support or hinder CAR-T cell function. Notably, clinically relevant differences in baseline T cell differentiation, exhaustion status, and inflammatory exposure have been consistently reported across ALL, NHL, MM, and CLL, which has direct implications for manufacturing success, *in vivo* persistence, and therapeutic durability (196, 197). This is partly due to differences in the immune evasion strategies employed by these tumors as well as the cumulative impact of prior therapies on the patient’s immune system.

In ALL, T cells tend to retain a higher proportion of naïve and early memory subsets, which is associated with better expansion and persistence after CAR-T infusion (198). This phenotype is consistent with the high response rates and long-term persistence observed in CD19-directed CAR-T trials in pediatric and young adult ALL, representing one of the most clinically favorable settings for CAR-T therapy. In contrast, T cells from patients with NHL and MM often show signs of chronic activation and exhaustion, including upregulation of inhibitory receptors and a shift toward more differentiated effector phenotypes (199). These disease contexts are therefore characterized by intermediate CAR-T fitness constraints, where clinical strategies such as optimized leukapheresis timing, selection of less-differentiated T cell subsets, and improved culture conditions are increasingly implemented, while pharmacologic or genetic rejuvenation approaches largely remain preclinical. This makes it more challenging to achieve sustained responses in these patients, necessitating additional interventions to rejuvenate or enhance the fitness of collected T cells.

As previously mentioned, age-related inflammation can contribute to T cell dysfunction, and the presence of cancer may intensify inflammation, further accelerating T cell aging (200).

Numerous hematological malignancies, such as MM and CLL, show elevated levels of inflammatory cytokines, such as IL-6, both locally and systemically, which correlates with worse outcomes (201). MM, in particular, relies heavily on IL-6 during certain phases of its progression (201), and the presence of increased levels of the inflammatory cytokine IL-17 or T helper 17 cells in peripheral blood mononuclear cells (PBMCs) and the bone marrow environment is linked to worsened disease (202). In relapsed/refractory (R/R) MM, inflammation from the disease enhances cytokine signaling pathways and proliferation driven by lymphopenia, thereby accelerating immune senescence (203). This leads to a decrease in naive T cells and changes in the metabolism of the remaining T cells in patients with MM.

Moreover, research conducted by Suen and colleagues identified that T cell clones from MM patients displayed a senescent effector phenotype, underscoring the T cell dysfunction associated with MM. Clinically, these features translate into reduced CAR-T persistence and increased risk of early functional decline, motivating the exploration of manufacturing-stage interventions (e.g., cytokine modulation, signaling inhibitors, metabolic reprogramming), which are currently under early clinical or advanced preclinical investigation.

Similarly, CLL is associated with early T cell aging (204) and represents one of the most challenging indications from a T cell fitness perspective. Patients with CLL typically exhibit a low CD4:CD8 ratio, which correlates with a shorter overall survival rate (204). Additionally, T cells from these patients show inherent functional impairments; when stimulated *in vitro*, their proliferation is significantly lower than that of T cells from age-matched R/R MM patients or young adults with ALL (205). Although CD8 T cells in patients with CLL can produce cytokines such as IFNγ and TNF, their cytotoxic function is hindered by issues such as improper granzyme localization and higher levels of PD-1 and other inhibitory receptors (64). This suggests that CLL contributes to a state of exhaustion in which T cells can recognize tumor antigens but are unable to effectively manage the disease. From a therapeutic modulation standpoint, most strategies in CLL including checkpoint blockade, cytokine pathway inhibition, epigenetic modulation, and signaling “rest” approaches remain largely preclinical or investigational, with limited clinical validation to date.

Acute myeloid leukemia (AML) represents a clinically important and biologically distinct context in which T cell fitness constitutes a profound limiting factor (206, 207). Although several early-phase CAR-T trials are ongoing in AML, no CAR-T therapy has yet been approved for clinical use in this indication, underscoring the substantial barriers to successful translation (208). In AML, T cells frequently exhibit baseline features of advanced differentiation, functional exhaustion, and metabolic impairment, which undermine proliferative capacity and persistence even prior to CAR engineering (209, 210). Clinical failures/limited efficacy of immune checkpoint blockade in AML (211, 212) further illustrates that simple reversal of PD-1/PD-L1 signaling is insufficient to restore T cell function in this disease. This limited clinical benefit reflects the fact that Tcell dysfunction in AML is not driven by checkpoint signaling alone but instead arises from multilayered immunosuppressive mechanisms operating within the leukemic niche. Indeed, the AML tumor microenvironment is highly immunosuppressive, characterized by abundant Tregs, MDSCs, and suppressive metabolites (e.g., adenosine, kynurenine) that directly impair effector T cell activation, cytokine production, and proliferation (213, 214). Additionally, AML blasts exhibit downregulation of antigen presentation machinery and heterogeneous expression of target antigens, which limit both endogenous and engineered T cell targeting efficacy and promotes immune escape (215).

Together, these intrinsic and extrinsic constraints place AML among the most challenging hematological malignancies for CAR-T therapy, and most approaches to enhance T cell fitness in AML remain preclinical or investigational, including metabolic reprogramming, dual-antigen targeting, adaptation of culture conditions, and modulation of niche-derived suppressive signals (208, 216). This disease thus exemplifies the multifaceted barriers to CAR-T efficacy that extend beyond T cell exhaustion alone and illustrates the need for disease-specific therapeutic modulation strategies.

Collectively, these findings illustrate that cancer type and its associated inflammatory and treatment context impose distinct, disease-specific barriers to CAR-T efficacy, ranging relatively mild constraints in ALL to profound intrinsic dysfunction in CLL, advanced MM, and the highly immunosuppressive setting of AML. Systematic integration of clinically established optimization strategies with emerging preclinical T cell rejuvenation approaches will therefore be essential to tailor CAR-T manufacturing and treatment paradigms to each disease setting. A comparative overview of disease-specific CAR-T fitness constraints and modulation strategies is provided in **Table 2**.

**Table 2:** Disease-specific determinants of CAR-T cell fitness and therapeutic modulation strategies across major hematological malignancies.

Disease	Baseline T-cell phenotype at leukapheresis	Dominant barriers to CAR-T fitness	Clinically established modulation strategies	Preclinical / investigational modulation strategies	Key references
ALL	High proportion of naïve and early memory T cells; low exhaustion	Limited intrinsic dysfunction; lower chronic inflammatory burden	Optimized leukapheresis timing; standard manufacturing protocols with IL-7/IL-15–based expansion	Fine-tuning of culture conditions; metabolic optimization	(196–198)
NHL	Increased effector differentiation; moderate exhaustion; elevated inhibitory receptors	Prior chemotherapy exposure; chronic antigen stimulation; partial features of immune senescence	Enrichment of less-differentiated subsets; manufacturing optimization; improved costimulatory domains	Signaling modulation (e.g., transient CAR inhibition); epigenetic and metabolic reprogramming	(196, 198, 199)
MM	Reduced naïve T cells; senescent effector clones; metabolic dysfunction	Chronic inflammation (IL-6, IL-17); lymphopenia-driven proliferation (homeostatic proliferation after repeated therapies); immune senescence	Optimized leukapheresis timing; selection of early-memory subsets; improved culture systems	Cytokine pathway modulation; metabolic rewiring; epigenetic targeting; signaling “rest” approaches	(199, 199, 201, 203)
CLL	Severe functional impairment; low CD4:CD8 ratio; high PD-1 expression; defective cytotoxicity	Early T-cell aging; intrinsic exhaustion; impaired proliferation and granzyme trafficking	Limited efficacy of standard manufacturing optimization alone	Checkpoint blockade combinations; cytokine modulation; epigenetic reprogramming; CAR signaling control strategies	(204, 205)

ALL, Acute Lymphoblastic Leukemia; MM, Multiple Myeloma; CLL, Chronic Lymphocytic Leukemia; NHL, Non-Hodgkin Lymphoma.

### 9.4 Impact of prior hematological cancer treatments on CAR-T cell function

In addition to aging and cancer types, treatments can contribute to T cell dysfunction in patients with cancer. The phenotype and proliferative capacity of T cells in patients with hematological malignancies are also affected by prior treatment. Some therapies can harm the T cell pathways involved in the repair of DNA damage or protein turnover. Particularly, significant lymphopenia caused by drug or radiation exposure is considered a major factor contributing to T cell aging (217).

ASCT is a key therapy for hematological cancers. However, it perturbs the composition of the T cell compartment and causes significant metabolic changes, ultimately reducing T cell fitness (203). Changes in T cell composition caused by ASCT and other conventional therapies that induce lymphopenia are irreversible, resulting in permanent increases in central memory T cells and effector memory T cells in patients with MM (203). Moreover, the CD4/CD8 ratios can be affected by this treatment due to the increased proliferation of CD8 T cells in comparison to CD4 T cells in the lymphopenic environment following transplantation. In children and young adults receiving ASCT, the CD4:CD8 ratio typically returns to normal levels within a year after treatment (218), whereas in older adults, this ratio may take longer to normalize, if it ever does (219). Moreover, it has been shown that patients with B-cell malignancies typically have a lower CD4/CD8 ratio, fewer naïve T cells, and a more differentiated T cell phenotype that worsens after multiple chemotherapy lines (220, 221). In a recent preclinical study, Das et al. showed that cumulative chemotherapy cycles deplete naïve T cells in many pediatric cancer regimens, reducing the expansion potential of CAR-T cell therapy (221). In contrast, the chemotherapeutic drug bendamustine can lead to prolonged lymphopenia, increased Tregs, and enhanced function of MDSCs, negatively impacting CAR-T cell production and response in aggressive lymphomas (222–224). In MM, exposure to alkylating agents promotes more senescent CD8+ T cells, whereas immunomodulatory drugs improve T cell function (225). With the increase in immunotherapeutic options for the treatment of hematological malignancies, the ideal order for using such therapies, particularly when they target the same antigen, is still unclear and needs to be taken into consideration before any therapeutic decision. For example, patients with MM who experience disease progression after BCMA-directed therapies can still respond to BCMA-CAR-T cell treatment, but response rates and durability were suboptimal compared to those not treated with prior BCMA-directed therapies (226, 227). It has been previously shown that continuous exposure to bispecific antibodies, such as blinatumomab, which stimulates T cells via the TCR but lacks a co-stimulatory signal, may result in T cell exhaustion or anergy, potentially reducing the effectiveness of later CAR-T cell therapy (228). However, treatment-free intervals may help restore T cell phenotype and function (228). Studies in pediatric B-ALL showed that blinatumomab exposure is linked to a higher risk of early treatment failure and reduced CD19 expression in non-responders (229–231). Post-CAR consolidation with hematopoietic stem cell transplantation may be a beneficial approach to mitigate the risk of relapse in blinatumomab-exposed patients. Interestingly, the use of CD19 immunotherapies, such as tafasitamab and loncastuximab, prior to CAR-T cell treatment in patients with DLBCL does not preclude subsequent responses to CD19-directed CAR-T cell therapy (232, 233). Owing to their long half-life and possible competition for CD19 binding with CAR-T cells, a washout period may be advisable. Nevertheless, CAR-T cell therapy remains more effective than chemotherapy in patients with MM who have undergone extensive prior treatment and progress after receiving a bispecific antibody (234). Therefore, it is critical for successful CAR-T cell therapy to select treatments that can positively affect T cell fitness. For example, Bruton’s tyrosine kinase inhibitor (BTKi) ibrutinib administered to CLL patients during apheresis positively affects CAR-T cell response by reducing the immunosuppressive environment, enhancing TCM proportion, and T cell expansion, resulting in significant complete response (CR) rates (235). However, the timing of ibrutinib administration relative to leukapheresis is critical because its effect may vary based on the duration before or after treatment (236, 237). In r/r Mantle Cell Lymphoma (CML), the TARMAC trial (NCT04234061) showed promising results with time-limited ibrutinib (≥7 days before leukapheresis until MRD−) in combination with CAR-T therapy (238).

Moreover, some therapies, such as immunomodulatory drugs, have the potential to boost CAR-T cell efficacy after infusion and could be a promising approach in the treatment of hematological malignancies. In LBCL patients who relapsed after CAR-T cell treatment, lenalidomide administration showed improved overall survival compared to chemotherapy (239). Similarly, in MM, the treatment of patients with lenalidomide or pomalidomide post-CAR-T cell therapy has been shown to be safe and leads to CAR-T cell re-expansion, late-onset, and durable clinical responses in some patients (240).

In summary, integrating assessments of CAR-T cell activity and longevity along with monitoring residual disease can assist in forecasting disease relapses, enabling proactive measures before visible recurrence occurs.

Considering the individual effects of aging, chronic infections, cancer type, and treatment on T cell fitness, the cumulative impact of these factors on T cell fitness, particularly in older heavily treated patients, needs to be taken into consideration. Therefore, refined measures should be developed to assess T cell fitness to allow for a better design of T cell-based immunotherapeutics for older and heavily treated patients with hematological cancers contemplating cell therapies.

### 9.5 Impact of T cell harvest timing on CAR-T cell fitness in hematological malignancies

The manufacturing process of CAR-T cells is complex and subject to numerous variations at each step. These variabilities, such as the time of T cell collection and treatment, can affect the quality of the final product, ultimately impacting clinical outcomes. The timing of T cell collection, whether early in the disease course or after multiple lines of therapy, plays a significant role in determining the quality of the CAR-T product.

Early collected T cells tend to have a higher proportion of less differentiated subsets, such as TN and TSCM cells, which are crucial for the long-term persistence and efficacy of CAR-T cells (198). In contrast, T cells collected late in the disease course, after multiple rounds of chemotherapy, or in the setting of advanced disease, are often more differentiated, exhibit higher levels of exhaustion markers, and have reduced proliferative capacity (241). It has been shown that T cells collected at first relapse led to higher naïve T cell percentages and lower exhaustion markers, resulting in improved anti-CD19 CAR-T cell fitness in patients with r/r LBCL, showing a better overall response rate (ORR) (242). Similarly, in MM, T cells collected from patients early in myeloma therapy exhibit better fitness for CAR-T manufacturing, with a higher proportion of early memory CD8+ T cells, a better CD4/CD8 ratio, and greater proliferative capacity compared to those who were relapsed or refractory (243).

Moreover, it has been demonstrated that intensive previous chemotherapy lines are linked to a reduced response to CAR-T cell treatment in LBCL (244).

These observations underscore the importance of considering the timing of T cell collection as a key factor in optimizing the fitness of CAR-T products. In clinical practice, this may involve proactive monitoring of patients at high risk of relapse and initiating T cell collection before disease progression or before administering therapies that could compromise T cell fitness.

**Table 3** summarizes the key patient-specific factors that critically impact T cell fitness and, consequently, the efficacy of CAR-T cell therapy.

**Table 3:** Impact of patient-specific factors on T cell fitness relevant to CAR-T cell therapy.

Patient-specific factors	Mechanisms and consequences on T cell fitness	References
Chronic Inflammation	• Persistent inflammatory signaling drives T cell exhaustion and senescence • Decline in naïve T cell repertoire impairs antigen recognition and vaccine efficacy • SA-T cells secrete pro-inflammatory mediators, sustaining a chronic inflammatory loop • Elevated levels of TNF-α and IL-6 alter T cell activation thresholds, especially in the elderly • Eicosanoids such as prostaglandins negatively impact T cell function	(188, 189) (204) (188) (184) (190)
Aging	• Immunosenescence reduces TCR diversity, T cell proliferation and decline naïve T cell production • Upregulation of inhibitory receptors contributes to T cell dysfunction • Mitochondrial dysregulation affects T cell metabolic fitness • Epigenetic and transcriptional shifts disrupt T cell gene networks • Aged T cells show diminished response to infections and immunotherapy	(191) (199) (15) (117, 190) (194)
Cancer Type	• Distinct tumor microenvironments affect T cell exhaustion and memory phenotypes • ALL: Enriched in naïve and early memory T cells with better CAR-T expansion and persistence • NHL: Upregulation of inhibitory receptors and shift toward differentiated effector T cells • MM: IL-6 and IL-17-driven T cell senescence and altered metabolism and early T cell aging • CLL: Early T cell aging, low CD4:CD8 ratios and impaired cytotoxicity despite cytokine presence	(195) (198) (199) (202) (204)
Cancer Treatment	• Radiation exposure contributes to T cell aging • Chemotherapy and ASCT induce lymphopenia and naïve T cell loss • Bendamustine and alkylating agents increase T cell senescence, prolong lymphopenia, increase Tregs and enhance function of MDSCs • Bispecific antibodies (e.g., blinatumomab) may cause T cell anergy or exhaustion • Ibrutinib improves T cell expansion when administrated during leukapheresis • Post-infusion lenalidomide/pomalidomide can rejuvenate CAR-T cells in some cases • Immunomodulatory drugs improve T-cell function	(217) (217) (223) (228, 229) (236, 238) (233, 245) (225)
Time of T Cell Collection	• Early collection ensures a higher frequency of TN and TSCM subsets • Late collection after multiple therapies yields exhausted and poorly proliferative T cells • First-relapse collections in r/r LBCL show better response and expansion • In MM, early-phase T cells maintain better CD4/CD8 ratios and metabolic fitness • Intensive prior therapies diminish CAR-T potency in B-cell non-Hodgkin’s lymphoma	(198, 243) (242) (244) (243) (246)

## 10 Conclusion and future perspectives

This review reframes T cell fitness as an integrated property emerging from coordinated regulation of differentiation state, signaling architecture, metabolic–mitochondrial capacity, transcriptional stability, and patient-specific constraints. Rather than representing a static cellular attribute, T cell fitness constitutes a dynamic and quantifiable determinant of CAR-T cell persistence, functional durability, and therapeutic success. Importantly, this framework enables a transition from descriptive biology to practical clinical implementation. Systematic assessment of T cell fitness at key stages of the CAR-T workflow including leukapheresis, manufacturing, and pre-infusion product release, could inform risk stratification and guide rational intervention. Readouts such as memory subset composition, mitochondrial spare respiratory capacity, glycolytic flexibility, reactive oxygen species (ROS) burden, exhaustion-associated transcriptional signatures, and tonic-signaling profiles could be incorporated into standardized quality-control pipelines to identify products at risk of early dysfunction. At the therapeutic level, fitness-guided optimization could be implemented through modular strategies that are already clinically tractable, including cytokine conditioning during expansion, metabolic modulation of culture environments, receptor-level engineering (co-stimulatory domain selection, scFv tuning, tonic-signaling control), and short-term pharmacologic signaling “rest.” Integration of these approaches would allow CAR-T manufacturing to evolve from a uniform process toward an adaptive platform tailored to both product-intrinsic properties and patient-specific immune constraints.

Looking forward, systematic incorporation of T cell fitness metrics into prospective clinical trials and real-world manufacturing pipelines will be essential to establish predictive thresholds, define actionable intervention windows, and validate their impact on long-term clinical outcomes. This approach will support the development of precision CAR-T strategies in which product design, metabolic programming, and signaling architecture are deliberately aligned with host biology and disease context.

Within this framework, T cell fitness emerges not only as a mechanistic explanation for inter-patient variability in CAR-T efficacy, but also as a clinically actionable target that can be quantitatively assessed, engineered, and optimized to improve persistence, reduce relapse risk, and extend the therapeutic reach of CAR-T cell therapy across heterogeneous hematological malignancies.

Future research should therefore prioritize personalized intervention strategies that integrate metabolic reprogramming, enhancement of mitochondrial fitness, and modulation of co-stimulatory signaling pathways according to individual patient immune profiles. Such precision-guided approaches hold promise for improving CAR-T cell durability and therapeutic efficacy, ultimately translating into more consistent and durable clinical benefit for patients with hematologic cancers.

## Glossary

CAR-T cell: Chimeric Antigen Receptor T cell
ALL: Acute Lymphoblastic Leukemia
MM: Multiple Myeloma
CLL: Chronic Lymphocytic Leukemia
T-ALL: T Cell Acute Lymphoblastic Leukemia
r/r B-ALL: refractory or relapsed B-cell Acute Lymphoblastic Leukemia
HL: Hodgkin Lymphoma
NHL: Non-Hodgkin Lymphoma
DLBCL: Diffuse Large B Cell Lymphoma (DLBCL)
HRS: Hodgkin and Reed-Sternberg cells
FDA: Food and Drug Administration
TN: Naïve T cells
TCM: Central memory T cells
TEM: Effector memory T cells
TSCM: T stem cell memory cells (or Stem cell–like memory T cells)
TEMRA: Terminally Differentiated Effector Memory RA+ T cells
Tregs: T regulatory cells
TAMs: Tumor-associated macrophages
MDSCs: Myeloid-derived suppressor cells
CCR7: C-C chemokine receptor type 7
VSTs: Virus-specific T cells
MHC: Major histocompatibility complex
APCs: Antigen-presenting cells
TCR: T cell receptor
ITAMs: Immunoreceptor tyrosine-based activation motifs
TME: Tumor Microenvironment
CR: Complete remission
ASCT: Autologous stem cell transplantation
scFv: single-chain variable fragment
DD: Destabilizing domain
CRISPR/Cas9: Clustered Regularly Interspaced Short Palindromic Repeats/CRISPR-associated protein 9
OXPHOS: Oxidative phosphorylation
PD-1: Programmed cell death protein 1
PDL-1: Programmed death ligand 1
CTLA-4: Cytotoxic T-lymphocyte associated protein 4
TIM-3: T cell immunoglobulin and mucin domain 3
LAG-3: Lymphocyte-activation gene 3
BTLA: B- and T-lymphocyte attenuator
TIGIT: T cell immunoreceptor with Ig and ITIM domain
TNF-α: Tumor necrosis factor- α
IFN-γ: Interferon gamma
GzmB: Granzyme B
ASS: Argininosuccinate synthase
OTC: Ornithine transcarbamylase
PBMCs: Blood mononuclear cells
ASCT: Autologous stem cell transplantation
GLUT1: Intracellular glucose transporter 1
BET: Bromodomain and extraterminal protein
MAPK: Mitogen-activated protein kinase.
